# Leukocyte related parameters in older adults with metabolically healthy and unhealthy overweight or obesity

**DOI:** 10.1038/s41598-021-84367-7

**Published:** 2021-02-25

**Authors:** Shan-Shan Zhang, Xue-Jiao Yang, Qing-Hua Ma, Yong Xu, Xing Chen, Pei Wang, Chen-Wei Pan

**Affiliations:** 1 School of Public Health, Medical College of Soochow University, 199 Ren Ai Road, Suzhou, 215123 China; 2 The 3rd People’s Hospital of Xiangcheng District, Suzhou, 215134 China; 3 Department of Children Health Care, Affiliated Suzhou Hospital of Nanjing Medical University, No.26, Dao Qian Road, Suzhou, 215000 China; 4 Department of Health Economics, School of Public Health,, Fudan University, 130 Dong An Road, Shanghai, 200032 China; 5 Key Lab of Health Technology Assessment, National Health Commission of the People’s Republic of China (Fudan University), Shanghai, 200032 China

**Keywords:** Obesity, Diagnostic markers

## Abstract

It remains unclear whether leukocyte-related parameters could be used as biomarkers to differentiate metabolically unhealthy overweight/obesity (MUO) from metabolically healthy overweight/obesity (MHO). We aimed to examine the differences in the distribution of leukocyte-related parameters between older adults with MHO and MUO and the correlations of leukocyte-related parameters with individual components of metabolic abnormality. In the Weitang Geriatric Diseases Study on older Chinese adults aged 60 years or above, 404 individuals with MHO and 480 with MUO contributed to the analysis. Overweight/obesity was defined as body mass index (BMI) of 25 kg/m^2^ or more. MHO and MUO were discriminated based on the Adult Treatment Panel III (ATP III) criteria. Leukocyte-related parameters were assessed using an automated hematology analyzer. All leukocyte-related parameters except monocytes were elevated in MUO group compared with MHO group (all *P* < 0.05). The prevalence of MUO increased by 24% with each 10^9^/L increase of leukocytes after adjusting for confounders in the multiple-adjusted model (*P* < 0.01) and each unit elevation of other parameters except lymphocytes and monocytes were significantly associated with the presence of MUO (all *P* < 0.01). Trend tests revealed a linear trend for the association between MUO and all the leukocyte-related parameters (all *P* for trend < 0.05). Significant interactions between leukocyte-related parameters and sex on the presence of MUO were observed (all *P* value for interaction < 0.05). Higher leukocyte-related parameters were found in patients with MUO than those with MHO and were associated with higher prevalence of MUO which seems to be sex-dependent. Further studies are needed to see whether these parameters could be used as biomarkers for the screening or diagnosis for MUO in clinical or public health practice.

## Introduction

Overweight/obesity has been recognized as a major public health concern mainly affected by behavioral risk factors such as physical inactivity and dietary factors^1,2^. There are two subtypes of overweight/obesity, namely “metabolically healthy overweight/obesity” (MHO) and “metabolically unhealthy overweight/obesity” (MUO), which are differentiated by the absence or presence of metabolic disturbance^1,3^. The mortality observed with MHO was estimated to decrease by 30–50% compared to that with MUO^4^. Thus, figuring out cost-effective and easy-to-access biomarkers which could differentiate MUO from MHO, especially in older adults who were more vulnerable to MUO-related complications, is essential for health management of overweight/obesity in the communities.

Systemic low-grade and long-term inflammation has been suggested to play an important role in the pathological process of overweight/obesity. A study suggested that although some morbid obese were metabolically healthy, they also presented an altered rheological profile which is related with inflammation^5^. Previous studies have indicated that the concomitant inflammation activity was lower in MHO patients in contrast to those with MUO^1,6–8^. For example, a proteomics study observed pro-inflammatory levels were decreased and levels of anti-inflammatory biomarkers were increased in individuals with MHO^9^. Karelis et al.^10^ observed a lower favorable inflammation state in MHO subjects, as attested by low CRP levels, suggesting that lower inflammation could play a role in the protective profile of the MHO individuals, and this may be associated to a lower risk for cardiovascular disease. Phillips et al.^11^ found reduced inflammatory status increased the likelihood of metabolic health, particularly among obese subjects according to a cross-sectional study conducted on 2047 men and women aged 45–74.

However, previous studies assessing the role of chronic inflammation in MUO mainly focused on biomarkers like C reactive protein (CRP)^3,6,9^. CRP is widely known as an acute-phase response protein and thought as a bystander marker of inflammation, which did not play a direct role in the inflammatory process^12^. In addition, CRP is not a commonly measured biomarker in medical check-up programs among older adults, which limited its use from a public health perspective. Leukocyte-related parameters such as counts of total leukocytes, lymphocytes, monocytes, and neutrophils is another group of chronic inflammation biomarkers which are easy to obtain in medical check-up programs. There are limited studies comparing the distributions of leukocyte-related parameters between MHO and MUO groups among older adults. The lack of evidence highlights the pressing need to identify the specific leukocyte-related parameters which could differentiate the two subtypes of overweight/obesity, especially among older adults, among whom the harmful effect of chronic inflammation on overweight/obesity might be exaggerated^13^.

In this study, we examined the differences in the distribution of leukocyte-related parameters between older adults with MHO and MUO and the correlations of these parameters with each component of metabolic abnormality in a community-based study on older Chinese adults. These findings might have important implications for weight management and prevention of obesity-related cardiometabolic features among older populations.

## Materials and methods

### Study sample

This analysis is part of the Weitang Geriatric Diseases Study, which is a community-based study consisting of adults aged 60 years or older in the Weitang town of Suzhou, located in Eastern China. Detailed study protocol of the Weitang Geriatric Diseases Study has been described elsewhere^14–16^. In brief, each adult aged 60 years or older was invited to participate in the study based on local official records, while one was excluded if he or she was immigrant resident, lived there for less than six months or died. Of the 5613 eligible adults enrolled in the study, 4611 adults participated in the clinical examinations from Aug 2014 to Feb 2015 and 4579 participants with completed information contributed to the data collection ultimately. Among the 4579 participants, 884 individuals were defined to have overweight/obesity, which was defined as body mass index (BMI) of 25 kg/m^2^ or more based on the WHO criteria^17^.

This study was conducted following tenets of the Helsinki Declaration and the approval was obtained from the Institutional Review Board of Soochow University. Written informed consent was obtained from all the participants before data collection.

### Measurements of variables

Height of participants was measured in centimeters without shoes using a wall-mounted measurement tape and weight was observed in kilograms with light clothing through a digital scale. Blood pressure (BP) was measured at least 3 times after a rest interval of 5 min or longer by an automatic blood pressure monitor (Dinamap model Pro Series DP110X-RW, 100V2; GE Medical Systems Information Technologies, Inc., Milwaukee, Wisconsin, United States) and the average of the last two readings was calculated as the value of BP. Venous blood samples were collected after 12-h fasting for laboratory evaluations. The leukocyte-related parameters described as × 109 cells/L was obtained using an automated hematology analyzer (XT-4000i, SYSMEX, Japan) and levels of HDL-C, TG, and fasting plasma glucose were measured with a chemistry analyzer (Roche cobas c 501). Characteristics of social-demographic were collected by trained staff via a pre-designed questionnaire such as age, sex, education level and marital status. Variables of lifestyle such as smoking, alcohol drinking and tea consumption were self-reported.

### Definitions of MHO and MUO

Metabolically healthy and unhealthy overweight/obesity were discriminated using the Adult Treatment Panel III (ATP III) criteria^18^, in which the definition of MHO required individuals with overweight/obesity to have one or none of the following components: (1) systolic blood pressure (SBP) no lower than 130 mmHg or diastolic blood pressure (DBP) no lower than 85 mmHg or using antihypertensive drugs; (2) serum triglycerides (TG) of 1.7 mmol/L (150 mg/dL) or more or use of lipid-lower drugs; (3) blood high-density lipoprotein cholesterol (HDL-C) less than 1.04 mmol/L (40 mg/dL) in men and 1.29 mmol/L (50 mg/dL) in women; (4) fasting plasma glucose (FPG) of 7.0 mmol/L (126 mg/dL) or higher or previously diagnosed diabetes mellitus or use of medications for diabetes. If the participant with overweight/obesity had two or more of the above components, he or she was considered to have MUO.

### Statistical analysis

Continuous variables were described as mean ± standard deviation (SD) while categorical ones were presented as numbers and percentages. The differences of leukocyte-related parameters between MHO and MUO were examined using the Student’s t test (if normal distributed) or Mann–Whitney U test (if not normal distributed) after Shapiro and Wilk test conducted for estimation of normality distribution. Trend tests were used to examine the possible dose–response relationships of the proportion of MUO with levels of leukocyte-related parameters. Multivariable logistic regression models were fitted to estimate the associations between leukocyte-related parameters and the presence of MUO. Adjusted odds ratios (ORs) of higher quartiles compared with the lowest quartile for each unit increment of leukocyte-related parameters and their corresponding 95% confidence intervals (CIs) were calculated and presented. Only age and sex were adjusted in model 1. We additionally adjusted for potential confounders such as education and lifestyle variables (smoking, alcohol intake and tea consumption). The associations between leukocyte-related parameters and individual metabolic components in both MHO and MUO groups were investigated using a partial correlation analysis. A likelihood ratio test was performed to investigate the potential interaction effects between leukocyte-related parameters and sex on MUO and sex-stratified analysis was further performed if the interaction effect was statistically significant.

A two-sided *P* value of less than 0.05 was considered as a statistically significant level for all analyses. The procedure of statistical analysis was conducted using the Statistics Analysis System (version 9.4; Cary, NC) and SPSS version 21.0 (SPSS Inc., Chicago, IL, USA).

## Results

Among the 884 individuals with overweight/obesity, 404 (45.70%) were defined as MHO and the other 480 (54.30%) were defined as MUO. In general, MUO group had significantly higher SBPs (146.02 mmHg vs. 150.70 mmHg; *P* < 0.01), DBPs (88.26 mmHg vs. 90.03 mmHg; *P* < 0.05), FPGs (5.47 mmol/L vs. 6.22 mmol/L; *P* < 0.01) and lower HDL-Cs (1.14 mmol/L vs. 1.49 mmol/L; *P* < 0.01) as compared with MHO groups. (Table 1).

**Table 1:** Demographic, somatometric, and laboratory parameters in MHO and MUO individuals.

	Overweight/obesity			*P* value*
	Participants	Metabolic normality	Metabolic abnormality	
	(n = 884)	(n = 404)	(n = 480)	
Sex (women), %	47.40	39.10	54.40	< 0.01
Age, mean(SD), years	66.39 (5.29)	66.62 (5.46)	66.20 (5.14)	0.34
BMI, mean(SD), kg/m^2^	27.37 (8.03)	27.48 (11.64)	27.28 (2.20)	0.71
SBP, mean(SD), mmHg	148.56 (18.57)	146.02 (19.53)	150.70 (17.45)	< 0.01
DBP, mean(SD), mmHg	89.22 (10.89)	88.26 (11.43)	90.03 (10.35)	< 0.05
Fasting glucose, mean(SD), mmol/L	5.88 (1.42)	5.47 (0.69)	6.22 (1.45)	< 0.01
Triglycerides, mean(SD), mmol/L	1.63 (0.93)	1.13 (0.34)	2.05 (1.05)	< 0.01
HDL-cholesterol, mean(SD), mmol/L	1.3 (0.34)	1.49 (0.32)	1.14 (0.27)	< 0.01
Current smoking, %	26.70	28.50	25.20	0.11
Alcohol consumption, %	27.10	32.90	22.30	< 0.01
Tea consumption, %	41.00	45.80	36.90	< 0.01
Hypertension, %	66.20	54.70	75.80	< 0.01
Diabetes mellitus, %	12.00	0.70	21.50	< 0.01
No formal education, %	44.50	44.60	44.40	0.92

*SD* standard deviation, *BMI* body mass index, *SBP* systolic blood pressure, *DBP* diastolic blood pressure, *HDL* high-density lipoprotein.

*P values represent difference in characteristics by metabolic syndrome status based on analysis of t-test.

The distributions of leukocyte-related parameters by metabolic status as defined by the ATP III criteria are displayed in Table 2. In all participants with overweight/obesity, all leukocyte-related parameters except monocytes were elevated in MUO group compared with MHO group (all *P* < 0.05). Sex-stratified analysis revealed that the pattern of associations between leukocyte-related parameters and MHO/MUO was different between men and women. The leukocyte count, neutrophil count and basophil count were significantly higher in MUO group than that in MHO group in men (all *P* < 0.05), while women with MUO were more likely to have increased count of neutrophils and eosinophils and decreased count of leukocytes (all *P* < 0.05) compared with their counterparts with MHO.

**Table 2:** Leukocyte-related parameters of study participants by metabolic status.

	Overweight/obesity			*P* value*
	Participants	Metabolic normality	Metabolic abnormality	
**All persons**	n = 884	n = 404	n = 480	
Leukocyte, mean (SD), × 10^9^/L	5.62 (1.44)	5.43 (1.54)	5.79 (1.33)	< 0.01
Lymphocyte, mean (SD), × 10^9^/L	1.76 (0.77)	1.70 (0.96)	1.81 (0.56)	< 0.05
Monocyte, mean (SD), × 10^9^/L	0.33 (0.13)	0.33 (0.12)	0.33 (0.14)	0.62
Neutrophil, mean (SD), × 10^9^/L	3.36 (1.04)	3.24 (1.03)	3.46 (1.04)	< 0.01
Eosinophil, mean (SD), × 10^9^/L	0.14 (0.13)	0.13 (0.13)	0.15 (0.13)	< 0.05
Basophil, mean (SD), × 10^9^/L	0.03 (0.02)	0.03 (0.02)	0.03 (0.02)	< 0.05
**Men**	n = 465	n = 246	n = 219	
Leukocyte, mean (SD), × 10^9^/L	5.68 (1.54)	5.49 (1.66)	5.90 (1.37)	< 0.01
Lymphocyte, mean (SD), × 10^9^/L	1.82 (0.92)	1.66 (1.14)	1.79 (0.56)	0.13
Monocyte, mean (SD), × 10^9^/L	0.35 (0.15)	0.35 (0.12)	0.36 (0.17)	0.42
Neutrophil, mean (SD), × 10^9^/L	3.42 (1.05)	3.30 (1.02)	3.55 (1.08)	< 0.05
Eosinophil, mean (SD), × 10^9^/L	0.15 (0.15)	0.14 (0.15)	0.17 (0.15)	0.08
Basophil, mean (SD), × 10^9^/L	0.03 (0.02)	0.03 (0.02)	0.04 (0.02)	< 0.01
**Women**	n = 419	n = 158	n = 261	
Leukocyte, mean (SD), × 10^9^/L	5.57 (1.32)	5.43 (1.54)	5.79 (1.33)	< 0.01
Lymphocyte, mean (SD), × 10^9^/L	1.81 (0.56)	1.76 (0.57)	1.83 (0.56)	0.20
Monocyte, mean (SD), × 10^9^/L	0.30 (0.10)	0.29 (0.10)	0.31 (0.09)	0.15
Neutrophil, mean (SD), × 10^9^/L	3.30 (1.03)	3.14 (1.04)	3.40 (1.01)	< 0.05
Eosinophil, mean (SD), × 10^9^/L	0.13 (0.10)	0.11 (0.09)	0.14 (0.11)	< 0.01
Basophil, mean (SD), × 10^9^/L	0.03 (0.02)	0.03 (0.02)	0.03 (0.02)	0.33

*P values represent difference in characteristics by metabolic syndrome status based on analysis of t-test.

Table 3 shows the multivariate-adjusted associations of leukocyte-related parameters with the presence of MUO in logistic regression models. The prevalence of MUO increased by 24% with each 10^9^/L increase of leukocytes after adjusting for confounders in the multiple-adjusted model (*P* < 0.01). Besides, each unit elevation of other parameters except lymphocytes and monocytes were significantly associated with the presence of MUO (all *P* < 0.01). All ORs of higher quartiles compared with the lowest quartile for each unit increment of monocytes and eosinophils were significantly above 1.00 (all *P* < 0.05). Trend tests revealed that there was a linear trend for the association between MUO and all the leukocyte-related parameters (all *P* for trend < 0.05).

**Table 3:** Adjusted Relationships of Leukocyte-related Parameters to the Prevalence of MUO.

	Age-and sex-adjusted		Multiple adjusted*	
	OR (95%CI)	*P* value	OR (95%CI)	*P* value
**Leukocyte, 10**^9^**/L**	1.24 (1.12,1.38)	< 0.01	1.24 (1.12,1.38)	< 0.01
Q1(≤ 4.72)	Ref		Ref	
Q2(4.73–5.42)	1.46 (1.00,2.14)	0.05	1.42 (0.97,2.09)	0.07
Q3(5.43–6.35)	1.89 (1.29,2.78)	< 0.01	1.85 (1.25,2.73)	< 0.01
Q4(> 6.35)	2.45 (1.66,3.63)	< 0.01	2.48 (1.66,3.68)	< 0.01
P for trend	< 0.01		< 0.01	
**Lymphocyte, 10**^9^**/L**	1.26 (0.99,1.59)	0.06	1.25 (0.98,1.59)	0.08
Q1(≤ 1.37)	Ref		Ref	
Q2(1.38–1.67)	1.44 (0.99,2.10)	0.06	1.44 (0.98,2.11)	0.06
Q3(1.68–2.06)	1.76 (1.20,2.57)	< 0.01	1.83 (1.24,2.70)	< 0.01
Q4(> 2.06)	2.01 (1.36,2.96)	< 0.01	2.04 (1.38,3.03)	< 0.01
P for trend	< 0.01		< 0.01	
**Monocyte, 10**^9^**/L**	2.47 (0.78,7.79)	0.12	2.83 (0.90,8.89)	0.08
Q1(≤ 0.25)	Ref		Ref	
Q2(0.26–0.31)	1.77 (1.22,2.59)	< 0.01	1.72 (1.17,2.52)	< 0.01
Q3(0.32–0.37)	1.51 (1.03,2.23)	< 0.05	1.53 (1.04,2.26)	< 0.05
Q4(> 0.37)	1.53 (1.04,2.25)	< 0.05	1.56 (1.06,2.30)	< 0.05
P for trend	0.06		< 0.05	
**Neutrophil, 10**^9^**/L**	1.28 (1.12,1.46)	< 0.01	1.28 (1.12,1.47)	< 0.01
Q1(≤ 2.63)	Ref		Ref	
Q2(2.64–3.20)	1.45 (0.99,2.13)	0.06	1.41 (0.96,2.08)	0.08
Q3(3.21–3.87)	1.56 (1.07,2.29)	< 0.05	1.51 (1.02,2.22)	< 0.05
Q4(> 3.87)	2.21 (1.49,3.27)	< 0.01	2.22 (1.49,3.30)	< 0.05
P for trend	< 0.01		< 0.01	
**Eosinophil, 10**^9^**/L**	6.16 (1.89,20.08)	< 0.01	6.40 (1.94,21.17)	< 0.01
Q1(≤ 0.07)	Ref		Ref	
Q2(0.08–0.11)	1.70 (1.18,2.46)	< 0.01	1.62 (1.12,2.35)	< 0.05
Q3(0.12–0.18)	1.64 (1.13,2.40)	< 0.05	1.64 (1.12,2.41)	< 0.05
Q4(> 0.18)	1.98 (1.36,2.90)	< 0.01	2.01 (1.37,2.96)	< 0.01
P for trend	< 0.01		< 0.01	
**Basophil, 10**^6^**/L**	1.01 (1.00,1.02)	< 0.01	1.01 (1.00,1.02)	< 0.01
Q1(≤ 20)	Ref		Ref	
Q2(21–30)	1.21 (0.85,1.71)	0.29	1.17 (0.83,1.67)	0.37
Q3(31–40)	1.33 (0.91,1.96)	0.15	1.29 (0.87,1.91)	0.21
Q4(> 40)	1.58 (1.09,2.30)	< 0.05	1.64 (1.12,2.40)	< 0.05
P for trend	< 0.05		< 0.01	

*Adjusted for age, sex, smoking, tea consumption, alcohol consumption, and education level.

A partial correlation analysis revealed significant and positive correlations between neutrophil count and SBP (*r* = 0.10) and FBG (*r* = 0.11) in subjects with MUO (Table 4). Neutrophil count was positively correlated with SBP, but not with DBP. Positive correlations were found between TG and counts of leukocytes, neutrophils, eosinophils and basophils. Eosinophil count was negatively correlated with HDL-C (*r* = − 0.10).

**Table 4:** Correlations between metabolic risk factors and leukocyte-related parameters from MHO and MUO elders.

	BMI	SBP	DBP	Triglyceride	HDL-cholesterol	Glucose
**MHO**						
Leukocyte, mean (SD), × 10^9^/L	− 0.05	0.08	0.03	0.14*	− 0.05	0.02
Lymphocyte, mean (SD), × 10^9^/L	− 0.01	0.01	0.01	0.01	0.02	0.07
Monocyte, mean (SD), × 10^9^/L	− 0.01	− 0.03	0.00	0.10	− 0.05	− 0.10
Neutrophil, mean (SD), × 10^9^/L	− 0.06	0.11*	0.04	0.17*	− 0.07	− 0.04
Eosinophil, mean (SD), × 10^9^/L	− 0.04	− 0.09	− 0.03	0.13*	− 0.10*	0.10
Basophil, mean (SD), × 10^6^/L	− 0.05	0.06	0.03	0.12*	− 0.02	− 0.04
**MUO**						
Leukocyte, mean (SD), × 10^9^/L	0.02	0.09	− 0.06	0.02	0.00	0.10*
Lymphocyte, mean (SD), × 10^9^/L	0.03	0.02	− 0.04	0.07	− 0.01	0.03
Monocyte, mean (SD), × 10^9^/L	0.04	− 0.04	− 0.04	0.02	− 0.08	0.05
Neutrophil, mean (SD), × 10^9^/L	0.00	0.10*	− 0.04	− 0.02	0.01	0.11*
Eosinophil, mean (SD), × 10^9^/L	0.05	0.04	− 0.04	0.03	− 0.03	− 0.05
Basophil, mean (SD), × 10^6^/L	− 0.01	0.06	− 0.06	0.07	0.03	− 0.05

Adjusted for age, sex, smoking, tea consumption, alcohol consumption, and education level.

*BMI* body mass index, *SBP* systolic blood pressure, *DBP* diastolic blood pressure, *HDL* high-density lipoprotein.

*P < 0.05.

**Figure 1:**
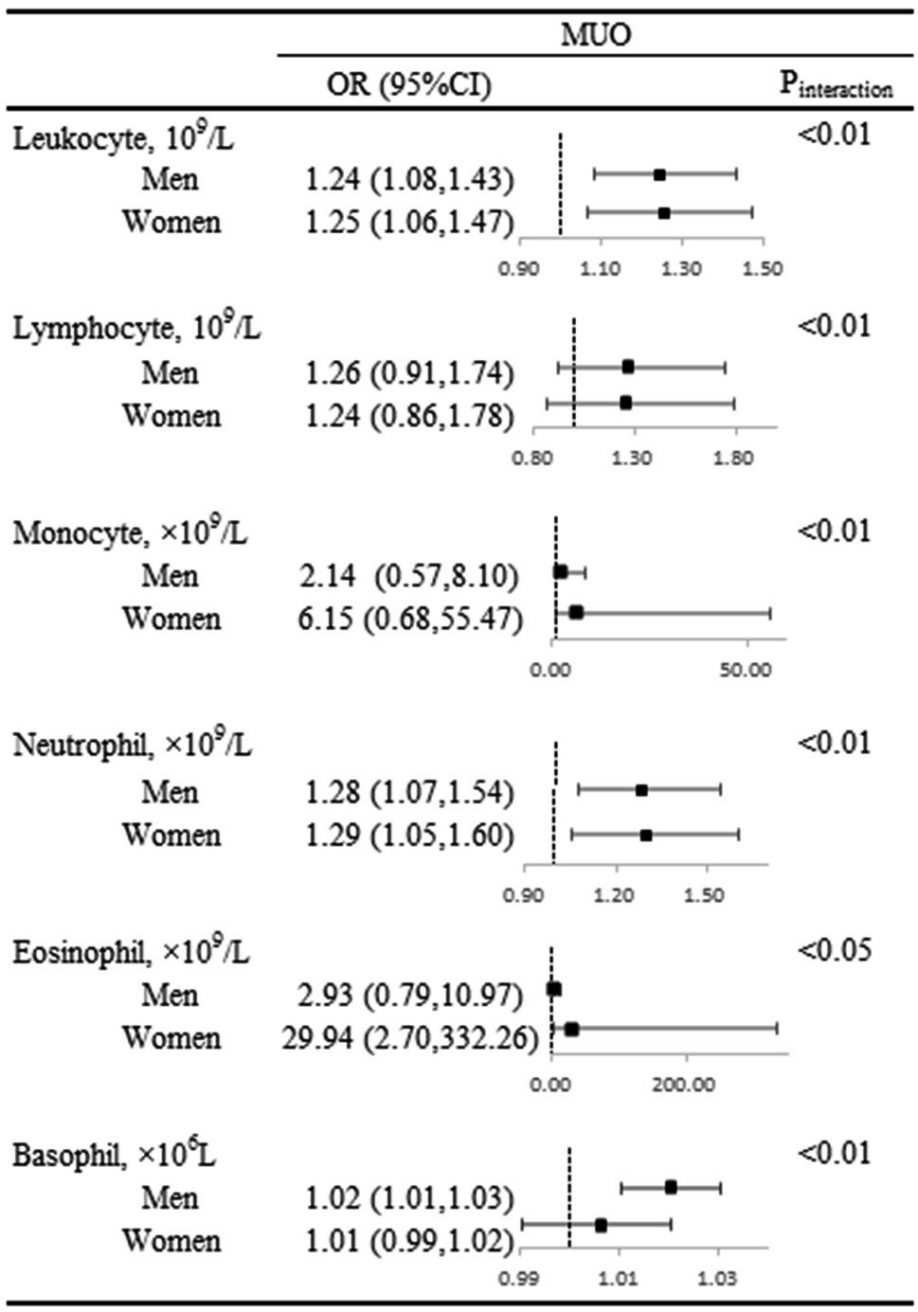
Subgroup analyses of the association between leukocyte-related parameters and MUO. Interactions between leukocyte-related parameters and sex on the MUO were tested by the likelihood ratio test with adjustment for the same variables as multiple models in Table 3 in addition to sex. Odds ratio and 95% CIs were showed by forest plot.

We further examined the relationship between leukocyte-related parameters and the presence MUO in men and women. Modest positive associations between leukocyte-related parameters except basophils and the presence of MUO were observed in both men and women. Furthermore, significant interactions between leukocyte-related parameters and sex on MUO were observed (all *P* value for interaction < 0.05; Fig. 1). Eosinophil count significantly elevated in MUO group in women (OR = 29.94, 95% CI [2.70, 332.26]) but not in men (OR = 2.93, 95% CI [0.79, 10.97], Fig. 1). On the contrary, basophil count was found to be significantly associated with a higher prevalence of MUO in men (OR = 1.02, 95% CI [1.01, 1.03]) whereas no significant association was observed in women (OR = 1.01, 95% CI [0.99, 1.02]; Fig. 1).

## Discussion

In this study, we observed that most of the leukocyte-related parameters were elevated in MUO group compared with MHO group, indicating that high levels of inflammation might be involved in MUO pathology. Moreover, the pattern of leukocyte-MHO/MUO associations might be different in men and women. Our study suggested that elevated leukocyte-related parameters might be related to a higher likelihood of MUO which seems to be sex-dependent.

Added to the above results, age and BMI were not significantly different between MHO and MUO group. There may be three possible reasons. Firstly, the subjects were all overweight or obese people over 60 years old, in which case there might be a correlation between age and metabolic status, but it was not inevitable, so did the relationship between BMI and metabolic status. Moreover, all participants were selected from the same town and thus they were likely to have certain homogeneity. Finally, the absence of such differences may also be due to the limitation of sample size.

Some previous studies have reported clinically significant differences in leukocyte-related parameters between MHO and MUO individuals and the findings seems to be inconsistent^6,8,19,20^. Our study showed a significant elevated level of leukocyte counts in the MUO group compared with their metabolically healthy counterparts, which was consistent with part of previous literatures^8,11,21^. Some other studies reported non-significant findings. For instance, Lynch et al.^20^ did not detect any differences in leukocyte count, but reported significantly higher levels of natural killer and cytotoxic T lymphocytes in MHO subjects which may protect against malignancy, infection and metabolic diseases. Wang et al.^22^ observed no significant differences in the counts of lymphocytes and neutrophils between MHO and MUO. A Mendelian Randomization study suggested the associations of blood eosinophil count and metabolic diseases may not be causal^23^. Moreover, contrary to our study, absolute counts of monocytes were higher in MUO subjects compared with MHO subjects as Christou et al.^5^ found in their study, while in their another study the difference appeared non-significant consistent with our results^24^. These conflicting findings may be explained by differences in methodological issues such as disparities in characteristics of the participants, inflammatory profiling and definitions of MHO/MUO^6^. Our study added novel evidence in this area by observing a dose–response relationship between leukocyte-related parameters such as leukocytes, neutrophils, eosinophils and basophils and the presence of MUO, which was not reported by previous studies.

The mechanism underlying the observations in the current study remains to be elucidated. Old adults tends to have elevated levels of free radicals and reactive oxidative species (ROS) and were more likely to be harmed by oxidative stress^25^. Leukocytes could promote oxidative stress and inflammation^26^ and have been demonstrated to play a role in insulin resistance (IR)^1,27^. IR is widely considered as the etiology of obesity and metabolic abnormality and may lead to higher gathering of inflammatory markers such as total leukocyte^28,29^. Moreover, shorter leukocyte telomere length (LTL) has been reported to be related to increased IR and oxidative stress^30^. Lubrano et al.^31^ reported a possible transition mechanism of MUO from MHO involved leukocyte-related parameters. Granulocytes and monocytes are primed by oxidized low density lipoprotein^32^, and then produce large amount of ROS and pro-inflammatory cytokines like IL-6 and TNF-α. Activated leukocytes in MHO individuals secrete excessive cytokines, thus prompting leukocyte migration into target tissues or organs for the initiation and maintenance of metabolic abnormality such as adipose tissue, pancreas and liver. Lipid oxidation may also induce adipogenesis and fat production by adipocytes^26^, creating vicious pathological loop of increased fat production and accumulation and metabolic disturbance mediated by leukocyte-related parameters. Additionally, leukocytes such as basophils were found to express receptors for leptin, a kind of adipokines secreted by adipocytes which affect energy expenditure, glucose and lipid metabolism and as well plays an important role in the pathophysiology of metabolic syndrome^33,34^.

The present study also found that the effects of leukocyte-related parameters on MUO significantly differed between men and women. First, a significant higher level of leukocyte count in MUO individuals was observed in men, while in women the count of leukocytes in MHO individuals was significantly higher than that of MUO individuals. Second, higher eosinophil count was significantly associated with higher prevalence of MUO in women but not in men, whereas basophil count was only found to be significantly associated with a higher prevalence of MUO in men. Gender difference in metabolic syndrome was also found but not discussed by others^35^. These inter-gender differences may be accounted for the differences in the behavioral and physiological variants between men and women. First, one reason for gender differences might be men had a higher frequency of smoking than women did in China, as cigarette smoking increases leukocyte count^36^. Second, the women subjects in our study were mostly postmenopausal. The body composition changes during menopausal transition including increased fat mass, leading to reduction in circulating adiponectin that is expressed in adipose tissue and correlated with many metabolic processes^37^. Premature telomere shortening have been discussed as potential mechanisms linking metabolic disorders, and postmenopausal women had decreased estrogen levels, which may prevent telomerase from being activated to catalyze the synthesis and extension of telomeres^38^. Particularly, shortened LTL might accelerate the leukocyte senescence and death and decrease leukocyte count. Third, the different variation of IL-6 and leptin by adipose tissue inflammation between men and women might also contribute to such sex-related association. A recent study demonstrated a positive association of adipose tissue inflammation with leptin and IL-6 specifically in men and a negative association of adipose tissue inflammation with adiponectin in women^39^. Fourth, sex-specific differences in mitochondrial function and free radical homeostasis could also contribute to the etiology of MUO^40^. Further studies are warranted in this area.

Considering the growing burden of overweigh/obesity worldwide and the increased death risk of MUO versus MHO^4^, our findings may have both public health implications. From a public health perspective, there is an urgent need to identify cost-effective biomarkers for MUO screening among older adults in rural communities, where the socioeconomic status is low. Our findings suggested that leukocyte-related parameters, a group of common and easy-to-access blood biomarkers, might be used by physicians and public health practitioners for differentiating MUO patients from MHO. On the other hand, we have to acknowledge that the finding of the study was preliminary and far from conclusive. Whether leukocyte-related parameters could be used as biomarkers for the screening or diagnosis for MUO in clinical or public health practice needs further clarifications.

To our best knowledge, this study was the first which comprehensively examine the differences in the distribution of a group of leukocyte-related parameters between older adults with MHO and MUO in a large-size community-based sample. However, several limitations in this study need to be noted. First, the study was cross-sectional and whether leukocyte-related parameters could predict the transition to from MHO to MUO could not be determined. Second, history of inflammation and infection of the participants were not collected, which may have altered the characteristics of inflammatory markers.

In conclusion, all leukocyte-related parameters except monocytes were significantly elevated in MUO individuals compared with MHO individuals and gender-specific patterns regarding the associations were observed. Further well-designed prospective studies are needed to assess the role of leukocyte-related parameters in the transition from MHO to MUO. The biological mechanisms underlying the observations should be elucidated as well.
